# Biochemical characterization of *Serpula lacrymans* iron-reductase enzymes in lignocellulose breakdown

**DOI:** 10.1007/s10295-019-02238-7

**Published:** 2019-11-16

**Authors:** Irnia Nurika, Daniel C. Eastwood, Timothy D. H. Bugg, Guy C. Barker

**Affiliations:** 1 Department of Agroindustrial Technology, Faculty of Agricultural Technology, Universitas Brawijaya, Malang, 65145 Indonesia; 2 Department of Biosciences, University of Swansea, SA28PP Swansea, UK; 3 Department of Chemistry, University of Warwick, CV47AL Coventry, UK; 4 School of Life Sciences, University of Warwick, CV47AL Coventry, UK

**Keywords:** *Serpula lacrymans*, Lignocellulose, Recombinant enzymes, Iron reductase, Fenton chemistry

## Abstract

Putative iron-reductase (IR) genes from *Serpula lacrymans* with similarity to the conserved iron-binding domains of cellobiose dehydrogenase (CDH) enzymes have been identified. These genes were cloned and expressed to functionally characterize their activity and role in the decomposition of lignocellulose. The results show that IR1 and IR2 recombinant enzymes have the ability to depolymerize both lignin and cellulose, are capable of the reduction of ferric iron to the ferrous form, and are capable of the degradation of nitrated lignin. Expression of these genes during wheat straw solid-state fermentation was shown to correlate with the release of compounds associated with lignin decomposition. The results suggest that both IR enzymes mediate a non-enzymatic depolymerisation of lignocellulose and highlight the potential of chelator-mediated Fenton systems in the industrial pre-treatment of biomass.

**Electronic supplementary material:**

The online version of this article (10.1007/s10295-019-02238-7) contains supplementary material, which is available to authorized users.

## Introduction

Biorefineries capable of the production of renewable chemicals from a range of suitable biomass feedstocks have been proposed as the basis for a biobased economy [1, 2]. Lignocellulosic biomass contains three major components: cellulose, hemicellulose, and lignin. Lignocellulosic biomass pre-treatment steps are required to make subsequent enzymatic digestion of each component efficient. Currently, such pre-treatments are expensive due to the cost of the energy or chemicals used [3, 4]. Developing a cost-effective method remains a significant challenge in the development of economic biorefineries.

In nature, fungi are the principle agents of lignocellulose degradation utilizing both enzymatic and non-enzymatic processes to convert biomass. White rot fungi produce ligninolytic enzymes such as class II peroxidases and laccases, which drive lignin depolymerisation and metabolism [5]. Brown rot fungi, derived from a white rot ancestry, have reduced enzymatic capacity to mineralize lignin and typically target the depolymerisation of hemicellulose and cellulose [6–8]. Studies into the mechanism used by different species of brown rot fungi and the subsequent effect on woody substrates have increased our understanding of the decay process [9–11]. Depolymerisation of lignocellulose is thought to be largely driven by a non-enzymatic chelator-mediated Fenton (CMF) system [10] that in the presence of Fe^2+^ generates reactive hydroxyl radicals [12–14]. Fe^3+^ reduction to Fe^2+^ in white rot fungi has been linked to cellobiose dehydrogenase (CDH) activity, extracellular hemoflavoenzymes produced by various lignocellulose-degrading fungi [15–17]. Fungus-derived oxalic acid, quinones, and others phenolic compounds produced through the degradation of lignin have also been implicated in the reduction of iron [18]. For example, 2,3 dihydroxybenzoic acid (DHBA) was reported as the chelating agent [12] released by the brown rot fungi *Gloeophyllum trabeum* [19] which is able to promote sustained iron reduction and generate reactive oxygen species (ROS). This suggests that the CMF reaction could become a self-sustaining process; however, it is unclear how the process is initiated.

Cellobiohydrolases (Glycoside hydrolases 6 and 7) associated with white rot endocelluloytic activity through the cleavage of β-1, 4 glycosidic bonds are absent or reduced in brown rots [8]. The Boletales brown rots *Serpula lacrymans* and *Coniophora puteana* retained one and two copies of GH6 cellobiohydrolase, respectively [8]. However, transcriptomic and proteomic analysis failed to detect the expression of this gene in *S. lacrymans* wood cultures [7, 20]. While the role of the *S.* *lacrymans* GH6 remains uncertain, a role for a chelator-mediated Fenton system in the depolymerisation of cellulose has been proposed for brown rot species [11]. Cellulose-targeted iron reduction, combined with substrate induction of iron-reducing phenolate biosynthesis, might explain the particular ability of brown rot fungi in the Boletales, such as the dry rot fungus *S.* *lacrymans*, to degrade crystalline cellulose without the presence of lignin [7]. Unusually, for the brown rots, two cellobiose dehydrogenase (CDH) genes were identified in *S.* *lacrymans*, but neither was expressed when the fungus grew on a wood substrate [7]. Two putative genes (number IR1 452187; IR2 417465) with similarity to the CDH iron-reductase domain were identified in the *S.* *lacrymans* genome [7]. These iron-reductase genes (IR1 and IR2) were postulated to play a role in lignocellulose decomposition, since IR1 contained cellulose-binding module-1 (CBM1) domain and was up regulated 122-fold when grown on wood compared to glucose medium [7].

The uncertainty in how *S. lacrymans* converts lignocellulose led us to investigate the iron-reductase genes to determine if they could be involved in the breakdown of lignocellulose and microcrystalline cellulose and, if so, their function. Recombinant *S. lacrymans* IR1 and IR2 proteins were produced using gateway plasmids cloned in *E.* *coli*. The function of these proteins was then tested for their iron reduction capabilities and the ability to release sugars and phenolic compounds through a putative chelator-mediated Fenton attack on lignocellulose. The results help to explain the particular ability of this brown rot fungus to degrade lignocellulose, and hence their potential as tools for the pre-treatment of biomass.

## Materials and methods

### Microorganism growth conditions and RNA extraction

The brown rot basidiomycete *Serpula lacrymans* S7 strain maintained within the culture collection of Warwick HRI (School of Life Sciences) was grown in the dark on 2% malt extract agar (MEA) plate at 20 °C for 3–4 weeks. This was used to inoculate 10 g of autoclaved wheat straw and cultured under solid-state fermentation (SSF) for 41 days. Samples were taken every 3 days and RNA extracted from 100 mg using a fast RNA Pro-Soil Direct Kit (MP Biomedicals). The purified RNA was quantified using spectrophotometer NanoDropTM ND-1000 and evaluated using the RNA 6000 Nano assay Kit (Agilent 2100 Bioanalyser). First strand cDNA was synthesized using the ThermoScript™ RT-PCR system (Invitrogen) following the manufacturers guidelines.

### The quantification of genes encoding iron reductase from *Serpula lacrymans*

The pattern of expression of the iron-reductase genes (IR1 and IR2) in wheat straw SSF cultures was determined through extraction of RNA and the use of QRT-PCR. RT-PCR amplification was performed in a 20 µl total reaction volume, using 1 µl of cDNA solution as template, 10 µl of Lightcycler 480 SYBR Green master mix (Roche Diagnostic Ltd), and 0.5 µM of each primer (Table 1). The amplification program consisted of an initial cycle (95 °C for 1 min), followed by 45 cycles of denaturation at 95 °C for 30 s, 60–62 °C for 1 min (temperature specific for each primer pairs) then extension at 72 °C for 30 s. A melting curve was obtained by performing 45 cycles at 95 °C for 1 min, 40 °C for 1 min, and 60 °C for 30 s and followed by 72 °C for 5 min. All reactions were done in triplicate in 384-well microtiter plates and a no-template control was included for each primer pair. Quantification of gene expression was determined relative to a standard curve for each target gene. Transcription of the iron-reductase genes (IR1 and IR2) was normalized and quantified by extrapolation to standard curves generated by plotting the logarithm of fluorescence versus cycle number for a serial dilution of cDNA template and to the housekeeping gene actin. The normalization of target gene and internal standard was carried out by correction with the endogenous control results [21].

**Table 1:** Primer sequences for IR1 and IR2 genes used for QRT-PCR

Name	Primer sequence 5′–3′ Forward	Primer sequence 5′–3′ Reverse	Length (bp)	*T*_m_ (°C)
Iron reductase (IR1)	GGCCTTGTCTTACCCCCTTTGTC	CCATAGTACCCCCAACGCTGAG	118	56.7
Iron reductase (IR2)	GCCTCACATTCCCTCCCGTATC	ATGGCCAGAGAACGAACAGTAAGC	147	56.7

### Cloning of iron reductase from the brown rot fungus *Serpula lacrymans*

To clone both genes (IR1 and IR2), primers were designed for the full length coding sequence (CDS), and the genes amplified from cDNA prepared from total RNA extracted from 41 days culture of *Serpula lacrymans* grown on wheat straw (Table 2). These were initially cloned using the TA cloning kit (Invitrogen) following the manufacture’s protocol. Sequence verification against the *S. lacrymans* genome was performed following Sanger sequencing using the ABI BigDye terminator V.1.1/3.1 seq Kit and alignment to the relevant accession number carried out (IR1 18816815; IR2 18813585). To optimize protein recovery in *E. coli*, the signal peptide was removed using an oligonucleotide primer designed to commence 60 base pairs from the start methionine. The amplified product included the appropriate adaptors for cloning into the GatewayTM system. Cloning into this system was then carried out using the standard protocol. The plasmid clone was then expressed in BL21 cells (www.invitrogen.com).

**Table 2:** Primer sequences for IR1 and IR2 genes as used for cloning into the gateway system

Name	Primer sequence 5′–3′
AttB1-IR1plusSP-F	AAAAAGCAGGCTTCatgGCTACAGCTTACTGCGATTC
AttB2-IR1minSTP-R	AGAAAGCTGGGTTCACAGGCACTGGCTATAATAC
AttB1-IR2minSP-F	AAAAAGCAGGCTTCatgGCTACTGCATACTGCGACTC
AttB2-IR2plusSTP-R	AGAAAGCTGGGTTCACAAGAGACTGAAAAAGTC
AttB1-adapter-F	GGGGACAAGTTTGTACAAAAAAGCAGGCT
AttB2-adapter-R	GGGGACCACTTTGTACAAGAAAGCTGGGT

### Production of recombinant protein

A transformed *E. coli* colony was inoculated into 10 ml LB medium containing the selective antibiotics (50 µg/ml carbenicellin and 34 µg/ml chloramphenicol) and grown overnight at 37 °C with shaking 220 rpm. 2.5 ml overnight culture was inoculated into 50 ml of prewarmed LB media (with antibiotics) on a shaking incubator (220 rpm for approximately 1.5 h), until the OD600 was 0.5–0.7 achieved. The transformants were induced using 0.4 mM of isopropyl-β-d-thiogalactopiranoside (IPTG) and incubated at 30 °C for an additional 5–6 h. The cells were harvested by centrifugation at 5000 rpm (20 min) for the SDS-PAGE analysis, resuspended in 1 ml lysis buffer containing 50 mM Tris–HCl pH 8; 1 mM EDTA pH 8,0; 1 mM tris2 carboxyethyl-phosphine (TCEP); 1 mM phenyl methylsulfonyl-fluoride (PMSF); 200 mM NaCl; deionized water (dH2O) and the cell pellet was frozen under liquid nitrogen and thawed in cold water. The cells were then sonicated for 6 × 10 s with 10 s pauses at 200–300 W and the lysate was centrifuged at 5000×*g* at 4 °C for 20 min. The soluble and insoluble fractions were tested for the presence of recombinant protein using a 12% SDS-PAGE gel.

### Purification of recombinant iron reductases

500 ml of LB culture was prepared for the purification of recombinant protein (IR1 and IR2) as described above. All protein purification was undertaken at 4 °C. The culture was centrifuged at 5000×*g* for 20 min at 4 °C and the pellet resuspended in lysis buffer containing 50 mM Tris–HCl pH 8; 1 mM EDTA pH 8.0; 1 mM tris2 carboxyethyl-phosphine (TCEP); 1 mM phenyl methylsulfonyl-fluoride (PMSF); and 200 mM NaCl. The cells were lysed using a combination of freeze–thaw and sonication method and then centrifuged at 13,000×*g* for 10 min at 4 °C and the supernatant was collected for the purification. The soluble fractions of recombinant protein (IR1 and IR2) were purified using Glutathione Sepharose 4B beads (GE Healthcare, UK) according to the manufacturer’s instructions. The crude cell extract was passed through a column pre-equilibrated with binding buffer PBS pH 7.5 (140 mM NaCl, 2.7 mM KCl, 10 mM Na_2_HPO_4_, and 1.8 mM KH_2_PO_4_). The columns were prepared according to the manual (Glutathione Sepharose 4B, 52-2303-00 AK). After extensive washing using binding buffer, the GST fusion proteins were eluted with elution buffer (50 mM Tris–HCl, 20 mM reduced glutathione, pH 8.0). Concentration of the recombinant protein was determined using the Bradford RC–DC protein assay (from Bio-Rad) using 1 mgml-1 BSA as the standard and absorption measured at 750 nm.

### Western blotting

Western blotting was carried out using the standard protocols [23]. The IR1 and IR2 recombinant proteins were transferred onto nitrocellulose membrane for 1.5 h and treated for 2–3 h at room temperature using 5% skimmed milk as the blocking agent. The membrane was then incubated overnight at 4 °C with primary antibody (monoclonal anti-GST antibody (SIGMA G-1160) at a dilution of 1:2000. The membrane was washed using three washes of PBST (Phosphate Buffer Saline with Tween 20) for 5–10 min each, then incubated with secondary antibody (Sigma-A4416 anti-GST antibody-peroxidase conjugate produced in mouse diluted in 1:10,000) for 2 h at room temperature. The blot was washed three-to-five times for 15 min using buffer PBST and then incubated with ECL (Enhance chemiluminesence) Western-blotting detection reagents (according to manufacture’s instructions from Amersham) for 5 min at room temperature before imaging.

### Determination of total soluble phenols released following SSF culture with the recombinant enzymes

Phenols were measured colourimetrically from wheat straw samples following inoculation with iron-reductase recombinant enzymes. This was carried out using the Folin–Ciocalteau method [22]. The concentration of phenols (per gram of substrate dry weight) was determined by reference to a standard curve prepared using gallic acid as the standard.

### Iron-reductase assay

A ferrozine-based colorimetric assay was used to detect release of Fe^2+^ by reduction of Fe^3+^. A modified method combining the approaches of [24] and [25] was developed to determine the reduction of iron using Ferrozine reagent [3-(2-pyridyl)-5,6-bis-(4-phenylsulfonic acid)-1,2,triazine] (Sigma). Using partially purified recombinant proteins (IR1 and IR2), the experiment was conducted in 96-well micro titer plates. A glutathione S-transferase green florescent protein (GFP) plasmid was also used expressed to provide a control protein for the experiment. 50 µl of crude extract/supernatant from soluble fusion protein of IR1 and IR2 cultures were combined with 0.1 mM FeCl_3_, 1 M acetate buffer pH 4.6, in the presence and absence of 50 µM 2,3 dihydroxybenzoic acid (DHBA). After 10 min incubation, 10 µM ferrozine reagent was added to the reaction. The absorbance was then measured at 550 nm using a spectrophotometer TECAN-Genious plate reader. Two readings were taken at 1 and 30 min.

### Nitrated lignin assay

This method was modified from [26] through the addition of Fe^3+^ as a substrate. 110 µl diluted nitrated organosolv lignin (prepared according to Ahmad et al. [26] was added to each well of a 96 well plate, followed by 40 µl 0.1 mM FeCl3, 10 µl of 50 µM 2,3-dihydroxybenzoic acid (2,3-DHBA), 30 µl recombinant protein of IR1 or IR2, and 10 µl 4 mM H_2_O_2_. The assay was monitored at 430 nm every minute for 20 min and carried out in quadruplicate. The whole plate was repeated as above but with 2,3-DHBA and/or H_2_O_2_ being replaced by deionized H_2_O. The bacterial lignin degrading enzyme *Rhodococcus jostii* DypB [27] and an *E. coli* GFP construct was used as positive and negative controls, respectively.

### Cellobiose dehydrogenase (CDH) enzyme assay

The CDH assay was slightly modified from Baminger [28] and [29]. The assay based on reduction of benzoquinone or dichlorophenolindophenol in the presence of cellobiose or lactose is recognized as a means of detecting CDH activity [30–32]. Recombinant enzyme activity was determined at room temperature using 0.1 M 2,6-dichlorophenol indophenol (DCPIP; Sigma-Aldrich) as an electron acceptor in two different buffers 50 mM sodium acetate buffer (pH 5) with cellobiose as the substrate. The reaction mixture (in a total volume 200 µl) containing: 100 µl of recombinant protein of IR1 or IR2; 40 µl of 0.6 mM cellobiose; 10 µl of 0.1 mM Fe^3+^ (Ferric chloride); 10 µl 2,3 dihydroxyl-benzoic acid 2,3 DHBA); 10 µl 4 mM H_2_O_2;_ and 10 µl 0.5 mM DPCIP. The CDH activity was measured by following a decrease in the absorbance of the electron acceptor DCPIP. The decrease in absorption of DCPIP was monitored using kinetic spectrophotometry (TECAN GENios) at 540 nm every minute from the first 60 s until 30 min. The whole assay was repeated without cellobiose, 2,3-DHBA, and recombinant proteins. All readings were taken in quadruplicate.

### Determination of the IR enzymes’ ability to release sugars from powdered wheat straw and cellulose (Avicel)

The total amount of reducing sugars released by *S.* *lacrymans*-derived iron reductases was quantified using the 3,5 dinitrosalicylic acid (DNS) method [33]. 1 ml of partially purified iron-reductase (crude extract) sample (IR1 and IR2) was prepared according to the method above. This was mixed with the two substrates (30 mg/ml) of Avicel-PH 101 (Sigma–Aldrich) and wheat straw powder. Samples were incubated for 24 h at 50 °C, pH 5.5 then allowed to cool to room temperature. An aliquot of the crude extract (250 μl) was taken and centrifuged for 1 min at 13,000 rpm. The experiment was carried out in the presence and absence of iron (0.1 mM Fe^3+^) and 4 mM H_2_O_2_ to test if a chelator-mediated Fenton system had a role in the release of sugar. 50 μM 2,3-DHBA was used as a positive control. Cellulase (1,4 β-d-glucan-,4 glucanohydrolase; Sigma–Aldrich C1184) at the same estimated concentration of the iron-reductase enzymes (0.312 mg/ml) was used as a positive control. The amount of reducing sugar released was measured at 540 nm in a plate reader (TECAN GENios) using four replicate and buffer without recombinant protein include as negative controls. The absorbances refer to the amount of total reducing sugars released as calculated from the glucose standard curve.

### Statistical analysis

The results were analyzed using analysis variance (ANOVA) in Genstat version 11 and the error bars represent LSD (*P* < 0.05).

## Results and discussion

### The quantification of genes encoding iron reductase from *Serpula lacrymans* and the relationship to phenols released

**Fig. 1:**
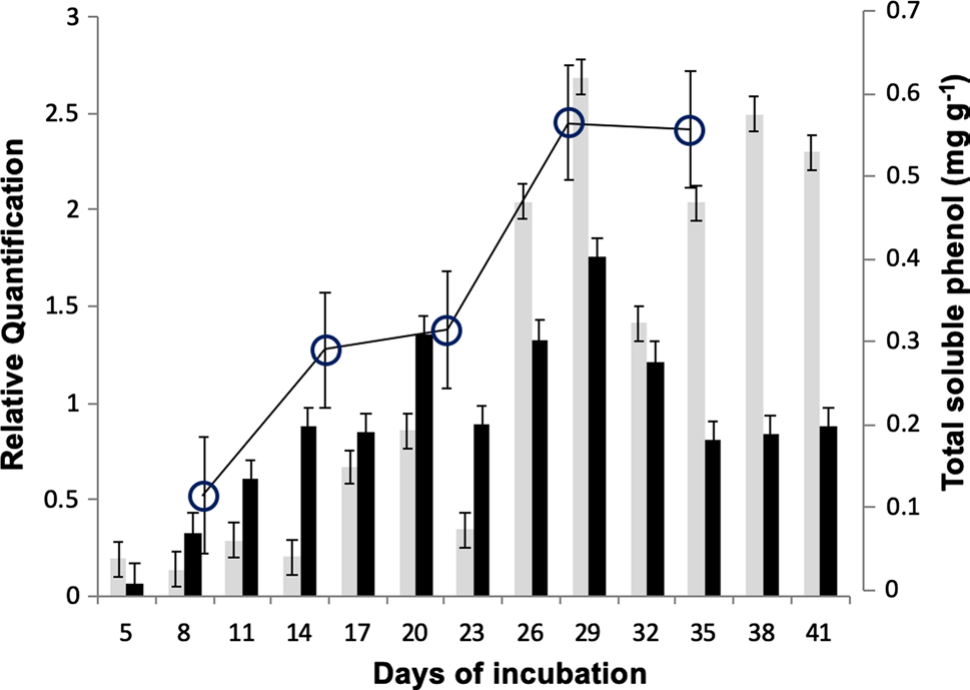
Relationship between the levels of IR gene expression and release of total soluble phenols during 41 day culture of *Serpula lacrymans* on straw. The error bars represent LSD (*P* < 0.05) derived from ANOVA. Relative quantification was measured against the expression of the actin gene (grey columns IR1; black columns IR2)

Quantification of the levels of gene expression of IR1 and IR2 was compared to the amount of aromatic (phenolic) compounds released during culture of *S. lacrymans* during solid-state fermentation (SSF) (Fig. 1). IR gene expression was shown to steadily increase up to 29 day incubation after which IR2 expression dropped, but IR1 expression remained high (Fig. 1). The increase in expression of both IR genes correlated with an increase in release of phenolic compounds supporting the hypothesis that both recombinant proteins are involved in substrate depolymerisation. Interestingly, in vivo IR2 expression was predominant during the early culture, but was overtaken by expression of IR1 with a cellulose-binding module CBM1 after 23 days (Fig. 1). These data support the hypothesis that the fungus generates an untargeted chelator-mediated Fenton system during initial substrate colonization to cause the initial disruption of the lignocellulose composite structure. This is followed by later hydroxyl radical generation targeted to exposed cellulose microfibrils by the binding of IR1 via a cellulose-binding module to maximise depolymerization of the primary carbon resource [7]. Temporal separation of lignocellulose depolymerization, where lignin-targeted oxidative reactions occurred before enzymatic depolymerization of hemicellulose and cellulose, has been reported in the brown rot *Postia placenta* [10]. To elucidate the functional role of the IR proteins in *S. lacrymans* decay mechanism, the IR genes were cloned.

### The expression and purification of the recombinant protein in *E. coli* (BL21)

**Fig. 2:**
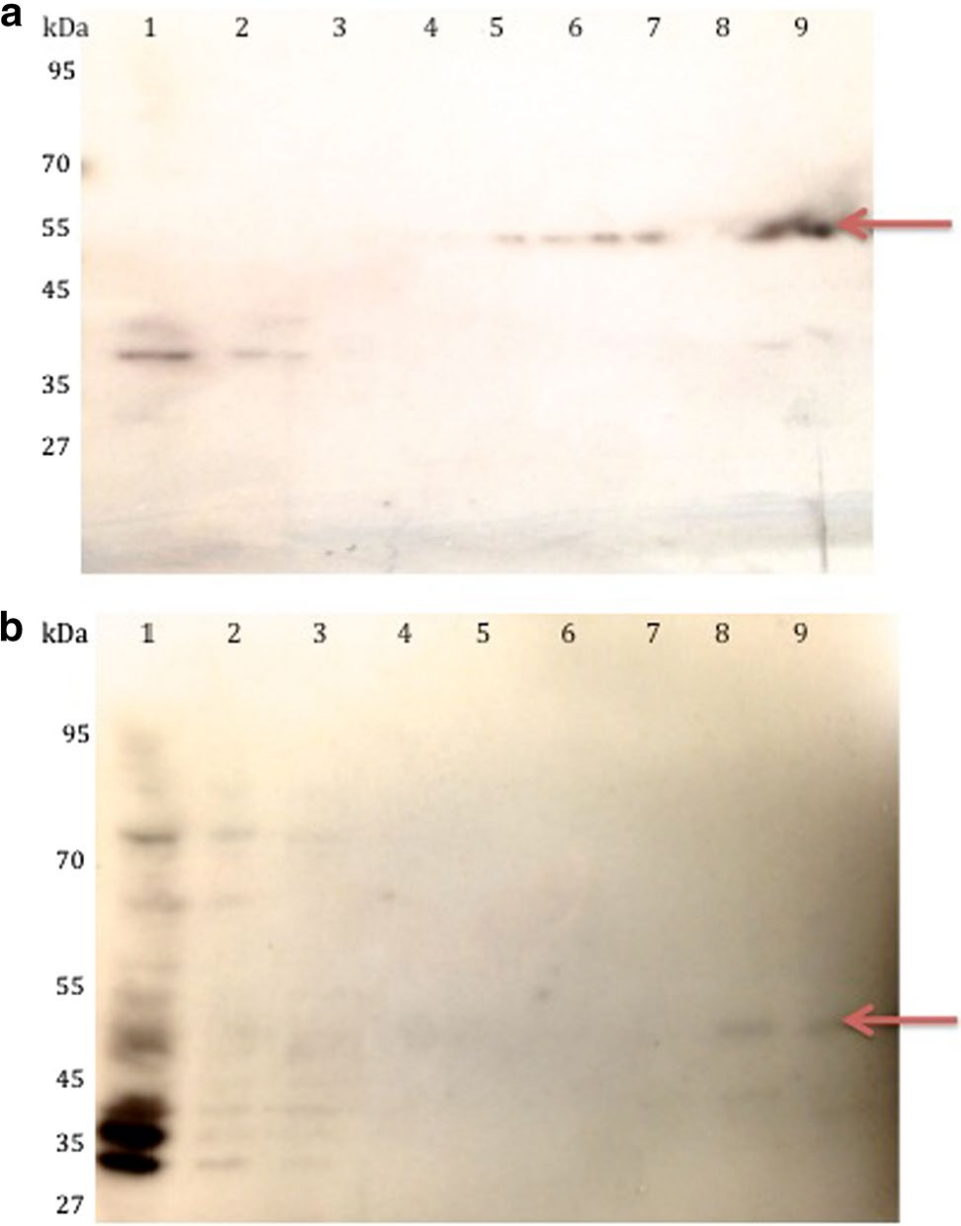
Bands at 55 kDa and 49 kDa, respectively, confirmed the presence of recombinant protein IR1 and IR2 by SDS-PAGE gels. The Western-blotting analysis of **a** IR1 and **b** IR2 purified using glutathione Sepharose 4B beads is shown. The arrows indicate the band corresponding to recombinant (**a**). IR1 (lane 1, crude extract; lane 2, flow through after initial column loading; lanes 3–4, wash fractions; lanes 5–7, elute fractions; lane 8, empty; lane 9, the elute fractions). **b** IR2 (lane 1, crude extract; lane 2, flow through after initial column loading; lanes 3–7, wash fractions; lanes 8 and 9, the elute fractions)

The cloned IR1 and IR2 genes were expressed in BL21 cells. A crude extract derived from the soluble fraction of the harvested cells following 5 h induction showed the highest production of the recombinant proteins at the expected size of 55 kDa and 49 kDa, respectively, for IR1 and IR2 (Fig. 2). After affinity purification using glutathione Sepharose 4B beads (GE Healthcare, UK), the expected proteins were observed and confirmed as GST fusion proteins using Western-blotting analysis with an anti-GST antibody (Fig. 2).

### Determination of the function of recombinant iron reductases (IR1 and IR2)

#### Iron-reducing capability

**Fig. 3:**
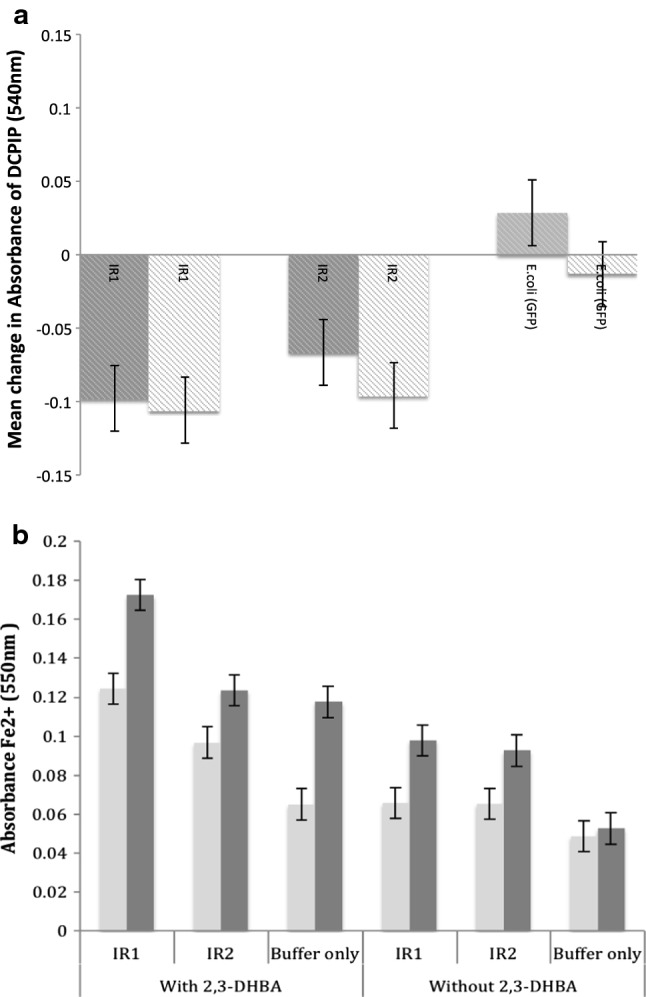
**a** Mean change of absorbance over 10 min observations of recombinant iron reductase (IR1 and IR2) from *Serpula lacrymans*. The CDH activity was determined by measuring on the decrease absorption of DCPIP at 540 nm. The negative level of samples (IR1 and IR2) showed the reduction of DCPIP absorbance as compared to the negative control (*E.* *coli* GFP). The grey columns represent the presence of 2,3-DHBA; the white columns represent the absence of 2,3-DHBA. The experiment conducted without cellobiose showed no change on the absorbance (data not showed). The error bars represent the least significant difference (LSD 5%). **b** Ferrozine forms a complex with ferrous iron that strongly absorbs light at 550 nm. The absorbance (550 nm) was measured following the addition of recombinant proteins IR1 and IR2 at 1 min (light grey columns) and 30 min (dark grey columns) observation on the form of Fe^2+^ both with and without the presence of 2,3-DHBA. The positive control was buffer with addition of 2,3-DHBA; negative control was buffer without the addition of 2,3-DHBA. The error bars represent the least significant different (LSD 5%) derived from ANOVA analysis

A hypothesis that IR1 and IR2 might have the capacity to reduce electron acceptors was suggested by the presence of a heme domain that is also present in CDH genes [7]. The presence of this domain is thought to allow CDH enzymes to oxidase the reducing ends of cellobiose, the main product of cellulose degradation, and reduce a wide range of electron acceptors including cytochrome c, dichlorophenolindophenol (DCPIP), benzoquinone, and Fe^3+^ [30–32]. Using a modified dichlorophenol indophenol (DCPIP)-based assay [28], the ability of iron reductases (IR1 and IR2) to reduce the electron acceptor was tested, with cellobiose as the substrate. The change in DCPIP absorbance was observed over 10 min after which all the DCPIP was assumed to be completely reduced, as no further change was observed in any sample. A significant reduction in DCPIP absorbance was apparent using both IR1 and IR2 [ANOVA analysis using LSD (*P* < 0.05)]. The negative controls (buffer alone and *E. coli* treatments) showed no reduction in DCPIP absorbance (Fig. 3a). 2,3-DHBA was also used as an iron chelating agent (see Fig. 3a) and as a control in the absence of IR proteins. The results show that the recombinant IR proteins are able to catalyse the reduction of Fe^3+^ to Fe^2+^ either with or without 2,3-DHBA (Fig. 3b). These data imply that the heme domain of IR1 and IR2 functions in a similar way to that found in CDH [29].

#### The ability of iron reductases to degrade nitrated lignin

**Fig. 4:**
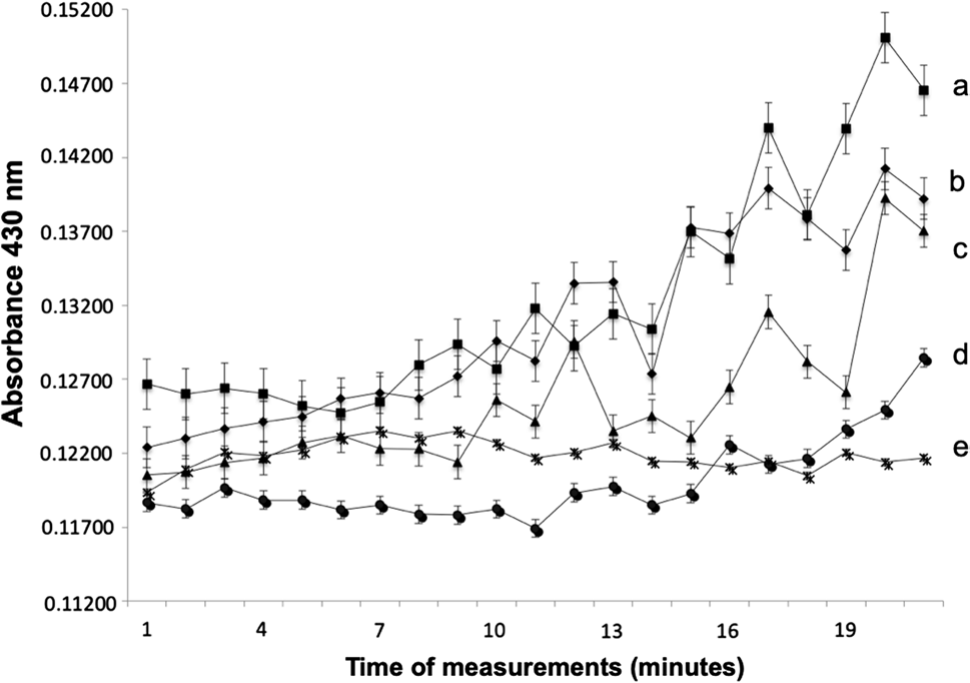
Changes in absorbance (430 nm) reflecting degradation of nitrated lignin by recombinant proteins (IR1 and IR2) with the addition of Fe^3+^ and H_2_O_2_; time dependent over 0–20 min. The assay was performed in the presence of 2,3-DHBA (a. square IR1; b. diamond IR2) and absence of with the addition of 2,3-DHBA (c. triangle IR1 and d. button IR2). The *E.* *coli* used as negative control (e. crossed), in the presence of Fe^3+^, H_2_O_2,_ and 2,3-DHBA. In the absence of hydrogen peroxide Fe^3+^ and (H_2_O_2_), no degradation was observed (data not shown). The error bars represent the least significant different (LSD) derived from ANOVA

The recombinant iron reductases were tested for their ability to degrade polymeric lignin using the nitrated lignin assay [26]. This assay measures the breakdown of lignin structure through the measurement of the amount of nitrated phenolic compounds released, as detected through an increase in absorbance at 420 nm. The activity was measured in the presence and absence of hydrogen peroxide (H_2_O_2_) and Fe^3+^ to confirm the involvement of Fenton chemistry in the degradation of nitrated lignin catalyzed by the presence of IR1 and IR2. *E. coli* GFP was used as a negative control and had little effect over the 20 min (Fig. 4), indicating its inability to degrade nitrated lignin. Both IR proteins had effects releasing significant amounts of phenolic compounds when compared to the controls. IR1 showed a greater activity than IR2 both in the presence and absence of 2,3-DHBA [34]. The depolymerisation of lignin is assumed to expose hemicellulose and cellulose in the substrate as well as the releasing of low molecular weight compounds such as quinones [34]. These could stimulate further iron cycling through a chelator-mediated Fenton system generating further hydroxyl radicals.

In the absence of iron (Fe^3+^) and 2,3-DHBA recombinant, IR1 and IR2 had no significant impact on the absorbance after 20 min (data not shown), indicating no decomposition of nitrated lignin. However, in the presence of Fe^3+^, a significant increase (*P* < 0.05, ANOVA using LSD) in the release of phenols was found using recombinant IR1 following 20 min incubation. IR2 on the other hand, only showed a significant difference when Fe^3+^ was present together with 2,3-DHBA. Without the 2,3-DHBA, there was no significant difference even after 20 min (Fig. 4). When 2,3-DHBA was added, it appeared to act synergistically with the IR genes, but showed no effect by itself (Fig. 4). In the absence of hydrogen peroxide (H_2_O_2_), IR1 and IR2 did not release phenolic products. The fact that H_2_O_2_ was required to support the idea that lignin degradation might occur via the Fenton reaction. This is supported by other research that implies the generation of hydroxyl radicals and/or superoxide radicals are required to break down lignocellulose and that the presence of iron, H_2_O_2,_ and chelating agent also plays a role [14, 35–39]. Figures showing the production of quinone (2,5 dimethoxy-1,4-benzoquinone) and oxalic acid during the culture of the fungus on straw support the evidence that these genes act as strong chelators with a function in non-enzymatic lignocellulose degradation (Supplemental materials).

### Release of reducing sugars from Avicel and wheat straw powder

**Fig. 5:**
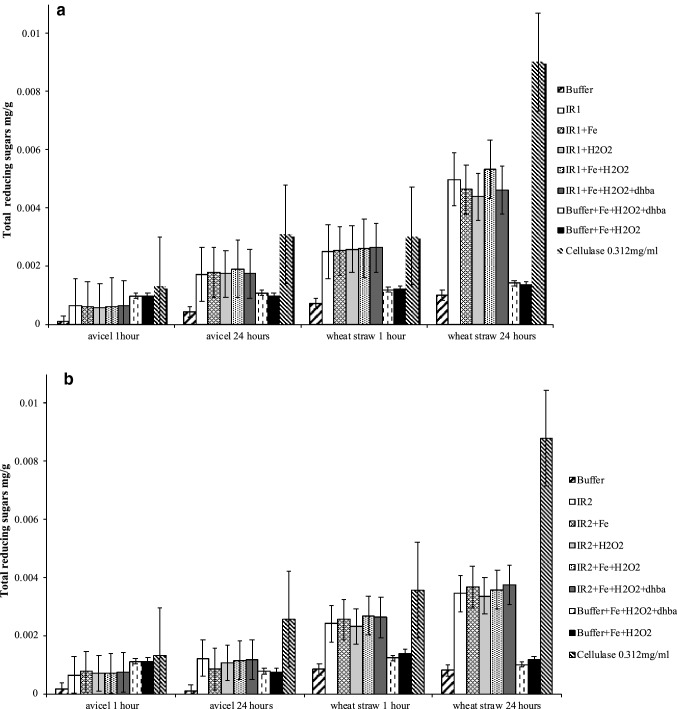
Amount of total reducing sugar released by recombinant protein iron-reductase IR1 (**a**) and IR2 (**b**), incubated on Avicel and wheat straw lignocellulose for 1 and 24 h at 50 °C in the presence and absence of H_2_O_2_, Fe^3+^, and 2,3-DHBA

To investigate whether the CMB1-containing IR1 was more efficient at substrate targeting, the partially purified recombinant iron-reductase (IR1 and IR2) proteins were assayed using the DNS assay [33], against microcrystalline cellulose (Avicel) and wheat straw powder for the release of reducing sugars. An increase in the release of reducing sugars was found with both protein extracts with both substrates compared to buffer only (control) after 24 h incubation (Fig. 5). When wheat straw powder was used as the substrate, there was a significant increase in the release of reducing sugars after 1-h incubation with IR1, IR2, or cellulase. The amount of sugars released was greater with IR1 when compared to IR2. Over 24 h incubation, the production of reducing sugars increased significantly using IR1 and cellulase. Lower levels of reducing sugars and greater variation between replicates were recorded in the presence of Avicel for both IR extracts and cellulase treatments. Both IR proteins increased sugar release after 24 h in comparison with the buffer only control, but gave a marginal increase compared to the iron, DHBA, and hydrogen peroxide controls. The difference between processed wheat straw and Avicel was not clear, but as the cellulase treatment gave similar results to the IR extracts, it is assumed that the greater grinding during wheat straw production increased the proportion of exposed cellulose filaments compared with Avicel. Unlike cellulase activity which is targeted to specific β(1,4) linkages within the cellulose polymer, the proposed IR activity is dependent on random cleavage of the polymer via radical attack. Increasing the number of exposed cellulose filament end through grinding of the straw into a powder could enhance the release of individual sugar units when exposed to IR activity. The incubation of IR proteins in the presence of iron, 2,3-DHBA, and H_2_O_2_ gave similar results to the recombinant proteins IR1 (Fig. 5a) and IR2 (Fig. 5b) alone. It is possible that the partially purified samples contained reactants that still enabled the proposed radical production and iron-cycling mechanism to occur. Alternatively, the proteins may contain an unidentified catalytic ability to disrupt β(1,4) linkages, possibly retained from their cellobiose dehydrogenase ancestry, even though no recognized glycosyl hydrolase domain is present in the derived IR proteins.

## Conclusions

While the data demonstrate that the potential of both IR proteins facilitates reactions that ultimately depolymerize the polymers found in lignocellulose, full elucidation of their role requires further study. The gene expression data clearly show high and differential expression of both IR genes during *S.* *lacrymans* growth on biomass, where the CMB1-containing IR1 has greater expression, as decay proceeds and cellulose becomes exposed. This does not mean that the enzyme is directly involved in cellulose depolymerization and instead might provide a greater localization of chelator-mediated cycling close to the substrate as cellulose binding becomes available (NB there is no known lignin-binding module). *S.* *lacrymans* has retained a GH6 cellobiohydrolase gene and exocellulases from the GH5 family are expanded and expressed during growth on plant biomass [7], suggesting that enzymatic cellulose depolymerization is present in the fungus. It is important to note that core iron-reductase activity of the IR proteins emerged from an existing cellobiose dehydrogenase enzyme, and this process involved further gene duplication event to create IR1 and IR2, whereby IR1 gained a CBM1, even though *S. lacrymans* showed lower CBM1 complement (23 total) compared with white rot species, e.g., 48 in *Phanaerochaete chrysosporium* [7]. Such events are unlikely to have occurred for a functionally redundant gene. Taken collectively, the data indicate that the IR proteins are utilized by *S. lacrymans* during lignocellulose decomposition to drive iron cycling most likely linked to a chelator-mediated Fenton mechanism targeted towards the disruption of lignin. Moreover, there is potential for enzymes such as IR1 and IR2 to drive cell-free chemically mediated biomass processing in a biorefinery setting.

## Electronic supplementary material

Below is the link to the electronic supplementary material.

Supplementary material 1 (DOC 396 kb)

## Abbreviations

GH: Glycosyl hydrolase
CBH: Cellobiose hydrolase
DHBA: Dihydroxybenzoic acid
CDH: Cellobiose dehydrogenase
CBM: Cellulose-binding module
CBD: Carbohydrate-binding domain
IR: Iron reductase
GST: Glutathione S-Transferase
LSD: Least significant difference
DCPIP: Dichlorophenolindophenols
DNS: Dinitrosalysilic acid
CMC: Carboxymethil cellulose
MEA: Malt extract agar
GFP: Green fluorescent protein
SSF: Solid-state fermentations
