# Supplementary material for: Biochemical characterization of Serpula lacrymans iron-reductase enzymes in lignocellulose breakdown
Source: J Ind Microbiol Biotechnol. 2019 Nov 16;47(1):145–54. doi: 10.1007/s10295-019-02238-7 (PMC6971154; doi:10.1007/s10295-019-02238-7)
Supplement: Supplementary file 1 — Supplementary material 1 (DOC 396 kb) [file 10295_2019_2238_MOESM1_ESM.doc]

**Electronic Supplementary Material**

**Biochemical characterization of *Serpula lacrymans* iron reductases enzymes**

**in lignocellulose breakdown**

Irnia Nurika^1^, Dan C. Eastwood^2^, Timothy D.H. Bugg^3^, Guy C. Barker^4*^

^1^ Department of Agroindustrial Technology, Faculty of Agricultural Technology, Universitas Brawijaya, Malang 65145, Indonesia, Tel: +62 341580106, email: [irnia@ub.ac.id](mailto:irnia@ub.ac.id)

^2^ Department of Biosciences, University of Swansea, United Kingdom, SA28PP Tel: +44 1792513003, email: [D.C.Eastwood@swansea.ac.uk](mailto:D.C.Eastwood@swansea.ac.uk)

^3^ Department of Chemistry, University of Warwick, United Kingdom, CV47AL, Tel: +44 2476523653, email : [T.D.Bugg@warwick.ac.uk](mailto:T.D.Bugg@warwick.ac.uk)

^4^ School of Life Sciences, University of Warwick, United Kingdom, CV47AL, Tel: +44 2476575135 email: [Guy.Barker@warwick.ac.uk](mailto:Guy.Barker@warwick.ac.uk)

*Corresponding author

Guy.barker@warwick.ac.uk

School of Life sciences, University of Warwick, Coventry, United Kingdom CV47AL. Tel: +44 2476575135

**Table of Content**

**1. Methods and Material**

1.1 Sample preparation

1.2 Solid state cultivation preparation

1.3 Quantification of oxalic acid production

1.4 Quinone production

**2. Results**

2.1 Sequencing of IR1 and IR2 using the ABI BigDye terminator V.1.1/3.1 seq Kit

2.2 The evidence of *Serpula lacrymans* released quinone (2,5 DMBQ) and oxalic acid

**1. Methods and Material**

**Sample preparation**

*Serpula lacrymans* S7 were supplied by Warwick Life Sciences collection and were grown on malt extract agar (MEA). Agar plugs of fresh mycelia were added to rye grain and grown at 20 ± 2° C to produce inocula (grain spawn) for cultivation. Rye grains (10 g) were previously placed into honey jars (250 ml), 13 ml water was added and autoclaved twice at 121º C for 1 hour.

**Solid state cultivation preparation**

Wheat straw was obtained locally from Warwick Life Sciences farm and chopped into small pieces (about 1-2 cm length), 10 g was placed into honey jars (250 ml) with 13 ml distilled water and autoclaved twice (121ºC for 1 hour). The prepared straw was inoculated with 1 g of grain spawn *S. lacryman*s and incubated at 20°C. Samples were collected following 0, 7, 14, 21, 28, 35, 42 and 49 days incubation.

**Quantification of oxalic acid production**

The level of oxalic acid was detected in all samples using HPLC and a wavelength of 210 nm with an organic acid column (Synergi 4U.Hydro.RT80A) with 0.005 N H_2_SO_4_ elulent at 0.5 ml min^-1^ (Clausen et al*.,* 2008). 60 µl of fungal aqueous extract were injected and compared against astandard oxalic acid curve prepared using 1.67, 3.13, 6.25, 12.5 and 25 mM concentrations. The retention time for oxalic acid was detected at approximately 10 minutes. The concentration of oxalic acid in fungal extract was determined by measuring the total area under the peak which was converted into mMs (Hunt et al., 2004).

**Quinone production**

In order to measure quinones (2,5 DMBQ), a modified method from Shimokawa et al., (2004) was used. 60 µl filtered supernatant (fungal aqueous extract), was applied to a C-18 HPLC column (Lichrospher 100; RP-18; 5µm). The column was eluted isocratically with water-acetonitrile-formic acid (80:20:0.1) as a carrier and run at 1.5 ml min^-1^ at ambient temperature. The absorbance was monitored at 280 nm and the quantitative results was obtained by calculating the product concentration using a 2,5 DMBQ standard curve and presented in mg unit. The retention time for 2,5-DMBQ using this system was 3.8 minutes

**2. Results**


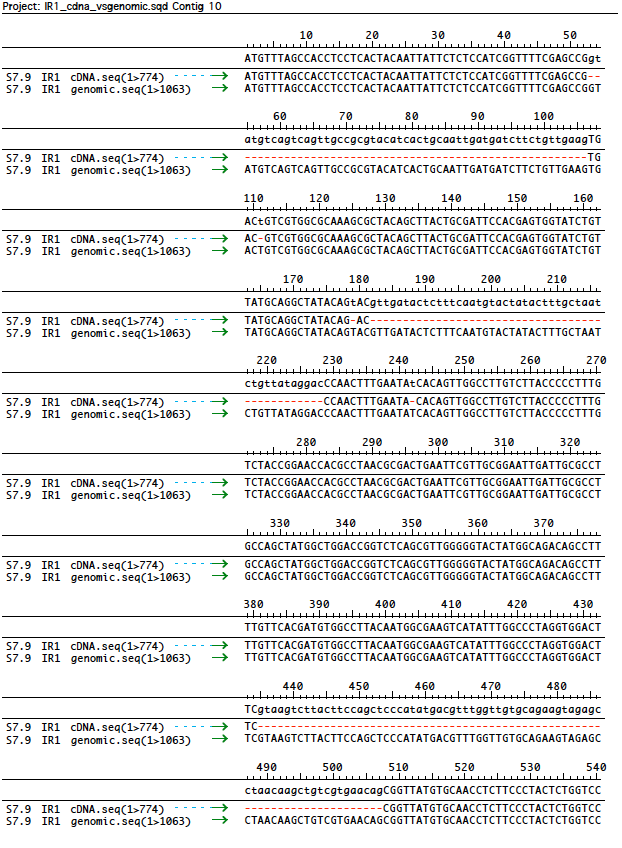
a **Sequencing of IR1 using the ABI BigDye terminator V.1.1/3.1 seq Kit**


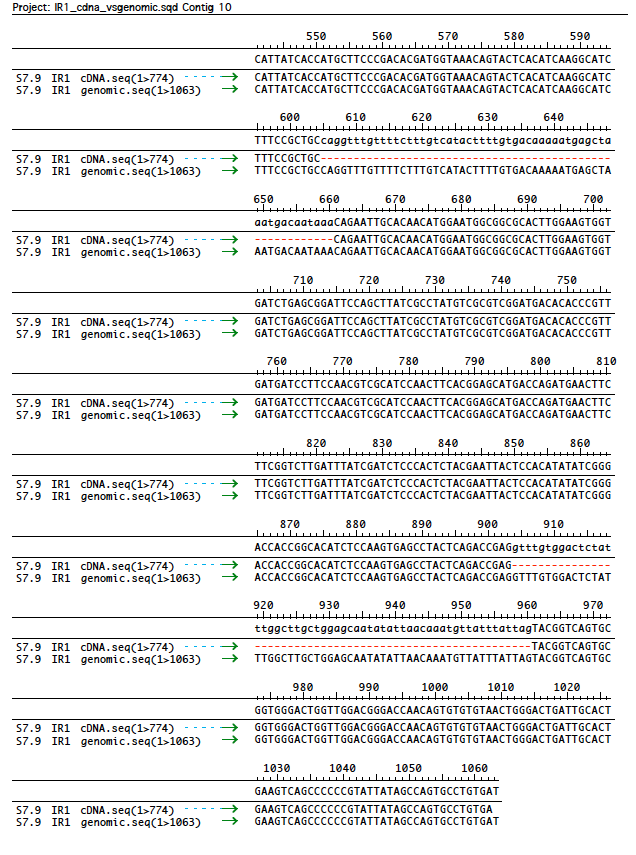


**b. Sequencing of IR2 using the ABI BigDye terminator V.1.1/3.1 seq Kit**


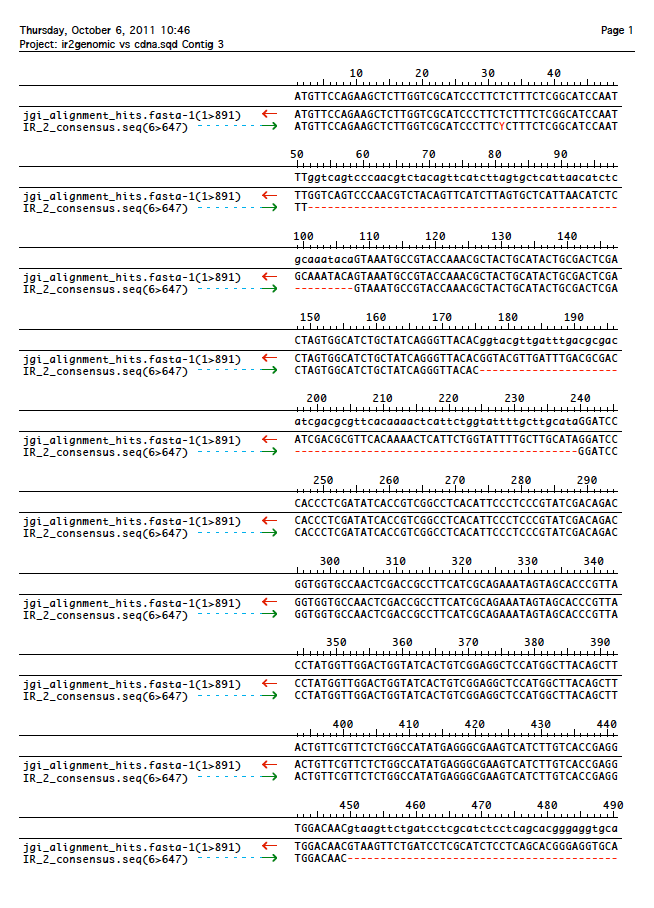


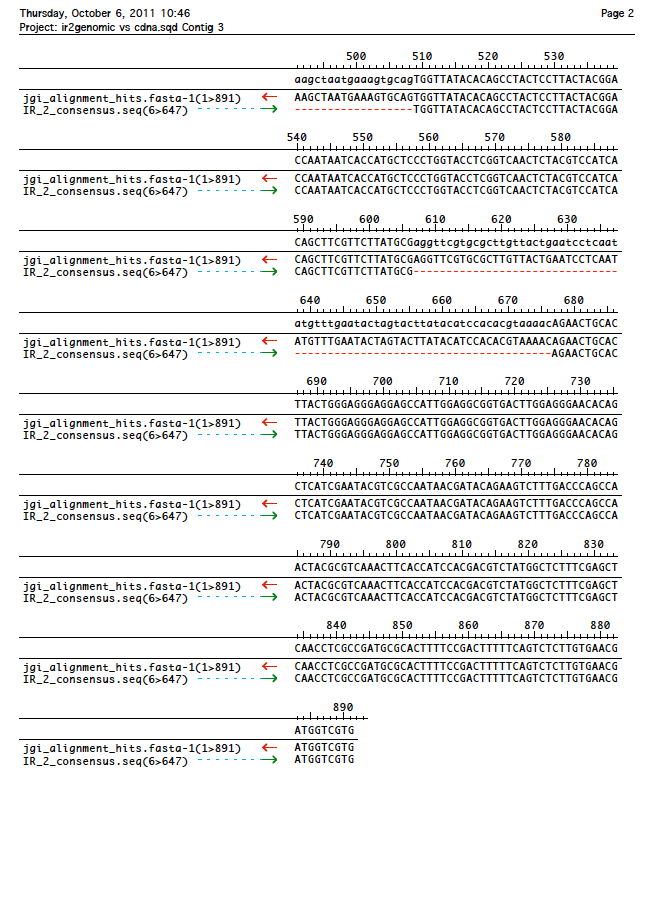


**2.2 The evidence of *Serpula lacrymans* released quinone (2,5 Dimethoxy-1,4-Benzoquinone) and oxalic acid**

In this study, the degradation of the cellulosic component has been shown to potentially be due to a non-enzymatic mechanism. This might well be due to the combined contribution of oxalic acid, quinone and other low molecular weight compounds. The role of oxalic acid for example reduces the pH of *S. lacrymans* wheat straw SSF culture which in turn modifies the biochemical composition of the wheat straw. By using the *S lacrymans* cultured in wheat straw, the extract of the cultured showed the production of quinone (2,5 DMBQ) and oxalic acid which was monitored at regular intervals over a period 49 days. A strong negative correlation (R^2^ = 0.98) between the quantity of oxalic acid and pH was found. The quinone (2.5-DMBQ) measured by HPLC showed the presence increased significantly over time and reached a maximum level of 28.69mM (0.48 mg g^-1^ straw) at 28 days, after which it declined to a level of 12.22mM (0.20 mg g^-1^ straw) on day 49.


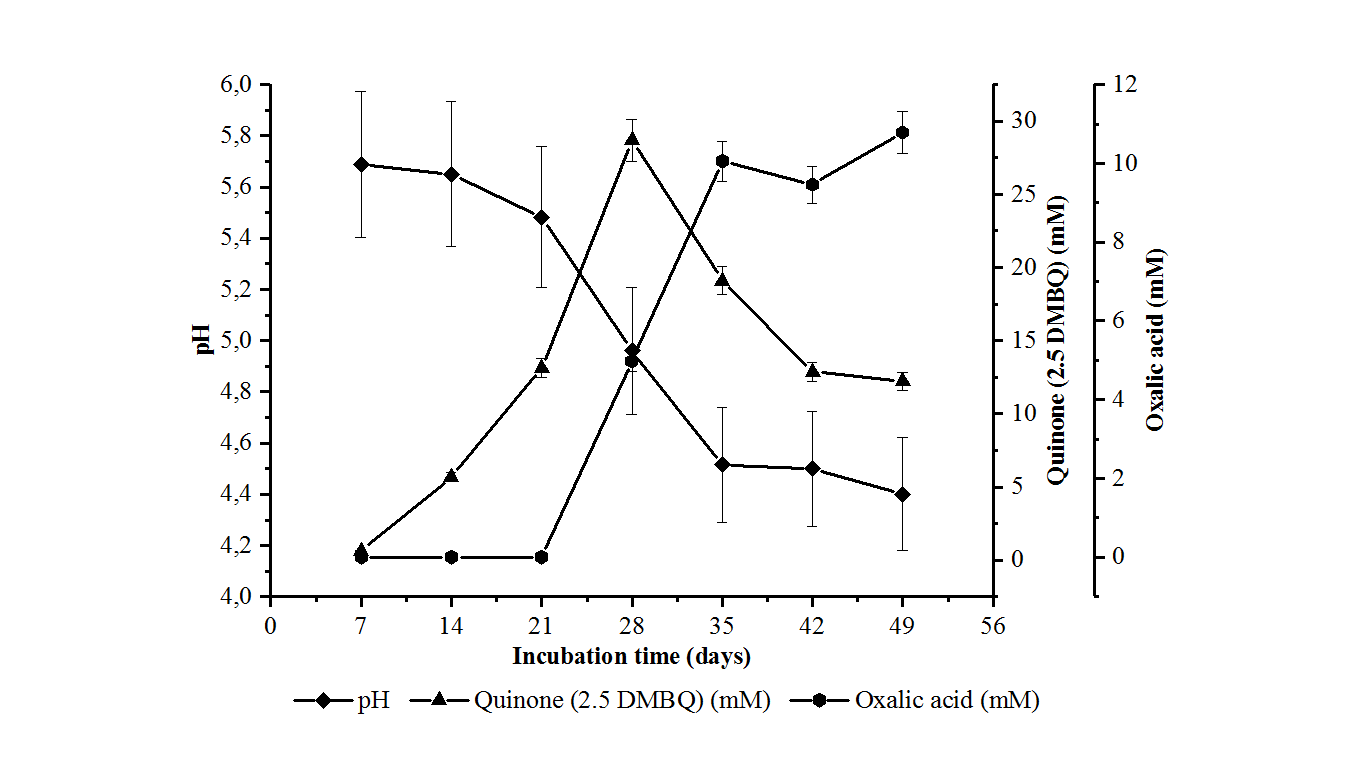


Figure 1. The relationship between pH, oxalic acid and released quinone by *Serpula lacrymans* cultured during a time courses of wheat straw SSF.

Oxalic acid as an extracellular metabolite thought to be involved in cellulosic breakdown due to its ability to increase pore size allowing penetration of fungal enzyme into the cellular structure (Dutton et al., 1993; Ritschkoff et al., 1995). The production and accumulation of extracellular oxalic acid by brown rot fungi is thought to be responsible for the rapid drop in pH (Green et al*.,* 1991). Oxalic acid not only reduces the pH but can also act as a strong chelator of Fe^3+^ (Fe oxalate complex) (Hegnar et al, 2019; Gamauf et al*.,* 2007). The most suitable pH for cellulose degradation was 4 (Xu and Goodell, 2001). This is similar to the final pH (pH = 4.4) measured after 49 days of culture with *S. lacrymans*. Moreover, It has previously been proposed that low molecular weight phenolic compounds (e.g. 2,5-dimethoxyhydroquinone/2,5DMBQ and 4,5 dimethoxycatechol/4,5DMC), might also function as catalysts for the Fenton reaction by reducing Fe^3+^ to Fe^2+^ (Kerem et al*.,* 1999; Hammel et al., 2002; and Shimokawa et al*.,* 2004). Two hydroquinones produced by *G. trabeum* (2,5-DMBQ and 4,5 DMBQ) are able to reduce Fe^3+^ oxalate complex. But, 2,5-DMBQ was found to be more effective than 4,5-DMBQ in the stimulation of the extracellular Fenton chemistry (Jensen et al*.,* 2001). 4,5-DMBQ was not detected within the current study with *S. lacrymans*. In this study the optimum quinone production (2,5-DMBQ) was found at 28 days before declining during subsequent incubation. Shimokawa et al*.* (2004), reported that the production of 2,5 DMBQ in *S. lacrymans* cultured in liquid media (broth culture) reached optimum levels of 90 µM within 2 weeks. The reduction in 2,5 DMBQ detected in this study after 28 days of culture suggests that *S. lacrymans* might reduce 2,5 DMBQ to 2,5 dimethoxy hydroquinone (2,5 DMHQ) as previously suggested (Shimokawa et al*.,* 2004).

**References**

Dutton, M.V., Evans, C.S., Atkey, P.T. and Wood, D.A. (1993). Oxalate production by Basidiomycetes, including the white rot species *Coriolus versicolor* and *Phanerochaete chrysosporium*. *Applied Microbiology and Biotechnology*, 39, 5-10.

Green, F., Larsen, M.J., Winandy, J.E. and Highley, T.L. (1991). Role of oxalic acid in incipient bronw rot decay. *Material Und Organismen*, 26, 191-213.

Ritschkoff, A.C., Ratto, M., Buchert, J. and Viikari, L. (1995). Effect of carbon source on the production of oxalic acid and hydrogen peroxide by brown rot fungus *Poria placenta*. *Journal of Biotechnology*, 40, 179-186.

Hegnar, O. A., Barry G., Claus F., Lars J., Nicole L., Keonhee K., Vincent G. H. E., Gry A., Anikó V., 2019 .Wood Science and Technology, Vol 53: 291-311

Gamauf, C., Metz, B. and Seiboth, B. (2007). Degradation of plant cell wall polymers by fungi. *Mycota*, 325-340.

Xu, G. and Goodell, B. (2001). Mechanisms of wood degradation by brown-rot fungi: chelator-mediated cellulose degradation and binding of iron by cellulose. *Journal of Biotechnology*, 87, 43-57.

Kerem, Z., Jensen, K.A. and Hammel, K.E. (1999). Biodegradative mechanism of the brown rot basidiomycete *Gloeophyllum trabeum*: evidence for an extracellular hydroquinone-driven fenton reaction. *FEBS Letters*, 446, 49-54.

Hammel, K. E., Kapich, A.N., Jensen, K.A. and Ryan, Z.C. (2002). Reactive oxygen species as agents of wood decay by fungi. *Enzyme and Microbial Technology*, 30, 445-453.

Jensen, K.A., Houtman, C.J., Ryan, Z.C. and Hammel, K.E. (2001). Pathways for extracellular fenton chemistry in the brown rot basidiomycete *Gloeophyllum trabeum*. *Applied and Environmental Microbiology*, 67, 2705-2711.

Shimokawa, T., Nakamura, M., Hayashi, N. and Ishihara, M. (2004). Production of 2,5-dimethoxyhydroquinone by the brown-rot fungus *Serpula lacrymans* to drive extracellular Fenton reaction. *Holzforschung*, 58, 305-310.
